# Dissecting the genomic regions, candidate genes and pathways using multi‐locus genome‐wide association study for stem rot disease resistance in groundnut

**DOI:** 10.1002/tpg2.70089

**Published:** 2025-08-11

**Authors:** H. V. Veerendrakumar, Hari Kishan Sudini, Bangaru Kiranmayee, Talwar Devika, Sunil S. Gangurde, R. P. Vasanthi, A. R. Nirmal Kumar, Sandip K. Bera, Baozhu Guo, Boshou Liao, Rajeev K. Varshney, Manish K. Pandey

**Affiliations:** ^1^ Center of Excellence in Genomics & Systems Biology (CEGSB) and Center for Pre‐Breeding Research (CPBR) International Crops Research Institute for the Semi‐Arid Tropics (ICRISAT) Patancheru India; ^2^ Department of Genetics and Plant Breeding, S. V. Agricultural College, Tirupati Acharya N. G. Ranga Agricultural University Guntur India; ^3^ CIMMYT‐China Wheat and Maize Research Center Shandong Agricultural University Taian China; ^4^ ICAR‐Indian Institute of Groundnut Research (IIGR) Junagadh India; ^5^ Crop Protection and Management Research Unit, USDA‐ARS Tifton Georgia USA; ^6^ Oil Crops Research Institute, Chinese Academy of Agricultural Sciences Wuhan China; ^7^ Centre for Crop and Food Innovation, WA State Agricultural Biotechnology Centre Murdoch University Murdoch Western Australia Australia

## Abstract

Stem rot, caused by *Sclerotium rolfsii* Sacc., is a devastating soil‐borne disease causing up to 80% yield losses in groundnut globally. To dissect the genetic basis of resistance, we evaluated a diverse minicore germplasm panel over 3 years in stem rot sick‐field conditions. Multi‐locus genome‐wide association study with the 58K single nucleotide polymorphisms (SNPs) Axiom_*Arachis* array genotyping identified 13 significant genomic regions associated with resistance across eight chromosomes with logarithm of the odds (LOD) scores ranging from 4.5 to 12.4 and *R* ^2^ values between 6.9% and 58%. Within these regions, 145 candidate genes were implicated, including wall‐associated receptor kinases, lucine‐rich repeat and NB‐ARC domain proteins, and peroxidase superfamily proteins. These genes orchestrate resistance through pathogen perception (e.g., receptor‐like kinases), direct inhibition (*R* genes), toxin detoxification, and activation of transcription factors driving protective compound synthesis for cell recovery. If these defenses are compromised, a hypersensitive response‐mediated apoptosis is triggered. Notably, resistance was exclusive to Virginia‐type groundnut. The identified candidate genes showed strong correlation with RNA‐seq data from stem rot‐infected plants, reinforcing their role in the transcriptional defense response. Three kompetitive allele‐specific PCR markers, namely, SnpAH00614 (on auxin‐related gene *AhSR001*), SnpAH00625 (on histidine triad protein gene *AhSR002*), and SnpAH00626 (on E3 ubiquitin ligase gene *AhSR003*), were validated, confirming their significant contribution to stem rot resistance. These markers may facilitate the development of stem rot‐resistant varieties through direct application in breeding programs through marker‐assisted selection.

## Core Ideas

- Marker trait associations (MTAs) and candidate genes associated with stem rot disease resistance have been identified through genome‐wide association study (GWAS).
- Pathway responsible for stem rot resistance has been dissected and understood.
- Identified genomic regions (single nucleotide polymorphisms [SNPs]) have been validated using allele‐specific markers.

**Abbreviations**
BP: biological process
CC: cellular component
DEGs: differentially expressed genes
GO: gene ontology
GWAS: genome‐wide association study
ICRISAT: International Crop Research Institute for the Semi‐Arid Tropics
KO: KEGG orthology
LOD: logarithm of the odds
MF: molecular function
MTA: marker trait association
PDI: percent disease incidence
PM: percent mortality
QC: quality control
QTL: quantitative trait locus
SNP: single nucleotide polymorphism
TF: transcription factor

## 1 INTRODUCTION

Groundnut (*Arachis hypogaea* L.) is a major oilseed crop and valuable food legume. It is globally cultivated over an area of 30.92 million ha, yielding approximately 54.27 Mt and an average productivity of 1755 kg per ha (FAO, 2023). Cultivated groundnut is a segmental allotetraploid with AABB genomes, derived from an interspecific hybridization between the diploid progenitors *Arachis duranensis* (A genome) and *Arachis ipaensis* (B genome) (Bertioli et al., 2016). Groundnut production is significantly impacted by various biotic and abiotic stresses, with biotic stresses posing major threats to yield. Among these, soil‐borne diseases are particularly problematic across growing regions (Jadon et al., 2015). Stem rot, caused by the soil‐borne fungus *Sclerotium rolfsii*, has an extensive host range of over 200 species and primarily infects plant parts that are coming in contact with soil. This pathogen is difficult to manage, as it produces sclerotia that can persist in soil for more than 25 years. Stem rot can lead to severe yield losses of 20%–80% by infecting the stem's crown region and subsequently damaging the entire plant (Kokalis‐Burelle et al., 2002). While fungicides provide some control, they are not a sustainable long‐term solution. Thus, host–plant resistance emerges as the most effective strategy, as it exhibits high genetic coefficient of variation for resistance (Veerendrakumar et al., 2024).

Current understanding of quantitative trait loci (QTLs) governing stem rot resistance remains limited, with several studies reporting distinct genomic regions. Initial mapping efforts (Dodia et al., 2019) revealed 44 epistatic QTLs, establishing the complex genetic architecture of this trait. Subsequent high‐resolution analysis (Luo et al., 2020) identified six major‐effect QTLs (qSR.A01‐2, qSR.A01‐5, qSR.A05/B05‐1, qSR.A05/B05‐2, qSR.A07/B07‐1, and qSR.B05‐1) with individual phenotypic variance explanation (PVE) > 10%, along with 12 significant additive × additive epistatic interactions. QTL‐seq approaches (Cui et al., 2020) precisely localized two resistance loci on chromosomes A01 (9% PVE) and A05 (13% PVE), demonstrating cumulative effects (21% PVE) consistent with additive inheritance patterns. The most comprehensive mapping to date (Agmon et al., 2022) detected 20 significant QTLs with genomic clustering on chromosomes A07, A03, B03, and B05, notably revealing the B05 hotspot (11.6%–21.7% PVE) as the most consistent genomic region associated with resistance.

Identifying resistance sources through reliable screening and incorporating these traits into breeding programs via genomic introgression are critical for developing resistant varieties. To facilitate this, a genome‐wide association study (GWAS) was conducted to identify genomic regions associated with stem rot resistance. This study employed a sick field screening across three seasons, utilizing a highly diverse collection of groundnut accessions (International Crop Research Institute for the Semi‐Arid Tropics [ICRISAT] minicore). The GWAS enables the identification of genomic regions or QTLs with high mapping resolution in natural populations. This technique leverages the historical recombination accumulated over generations, thereby enhancing precision (Vikas et al., 2022). By analyzing hundreds of thousands of genetic variants across diverse genomes, GWAS identifies statistically significant associations with specific traits or diseases. This method has produced numerous reliable associations across a wide range of traits, and in GWAS as sample sizes grow, the precision of identified variants is expected to increase. The outcomes of GWAS have applications in various domains, such as improving our understanding of the biological mechanisms underlying phenotypes, estimating heritability, and assessing genetic correlations (Uffelmann et al., 2021). Multi‐locus GWAS models are often preferred over single‐locus models for mapping genomic regions, as they estimate the effects of all markers simultaneously, leading to greater efficiency and reliability (Vikas et al., 2022). Despite advancements, relatively few QTL mapping studies have been conducted on stem rot, and there remains a substantial gap between the genomic regions mapped and their contribution to total phenotypic variance. To address this gap, precise analysis of genomic regions is needed through approaches such as QTL mapping, GWAS, and RNA sequencing (RNA‐seq). These analyses can help establish the cumulative contribution of specific genes to total phenotypic variance. In the present study, we employed a GWAS approach to identify relevant genes and compared the results with previously mapped regions identified in QTL mapping and RNA‐seq studies. The aim was to identify key genomic regions and major genes along with performing marker validation, which can be employed to achieve high level of resistance to stem rot disease in groundnut.

## 2 MATERIALS AND METHODS

### 2.1 Plant material

The study utilized a groundnut minicore germplasm collection from the ICRISAT Genebank, comprising 184 diverse accessions from 45 countries. This germplasm set, which includes various subspecies (*fastigiata* [96 accessions], *hypogaea* [88 accessions]) and growth habits like Decumbent‐1 (two accessions), Decumbent‐2 (22 accessions), Decumbent‐3 (27 accessions), Erect (99 accessions), and Procumbent‐1 (34 accessions), represents a highly diverse panel suitable for genetic studies. Phenotyping was conducted in a sick field at ICRISAT, Patancheru, Hyderabad, over 3 years (Veerendrakumar et al., 2024). Stem rot disease assessment involved measuring percent disease incidence (PDI) and percent mortality (PM) to quantify resistance levels.

**Core Ideas**

- Marker trait associations (MTAs) and candidate genes associated with stem rot disease resistance have been identified through genome‐wide association study (GWAS).
- Pathway responsible for stem rot resistance has been dissected and understood.
- Identified genomic regions (single nucleotide polymorphisms [SNPs]) have been validated using allele‐specific markers.

### 2.2 Sick‐field screening protocol

The groundnut mini‐core (184 accessions) was screened for stem rot resistance in a sick plot at ICRISAT, Patancheru (2016, 2017, 2022) using an alpha lattice design (two replications, 19 blocks, 10 plots/block). Plots (4 m × 1.5 m) were sown in single rows (30 cm × 10 cm spacing), with resistant (CS‐19) and susceptible (TMV2) checks. At 22 and 45 days after sowing, plants were inoculated with sorghum seed‐multiplied *S. rolfsii*. Disease progression (PDI, PM) was recorded at 30, 60, and 90 days after sowing using the formula PDI = (number of plants infected/total number of plants) × 100 and PM = (number of plants dead/total number of plants) × 100.

### 2.3 DNA extraction and genotyping using SNP array

The total genomic DNA was extracted from the young leaves of 25–30‐day‐old plants utilizing the Nucleospin Plant II kit (Macherey‐Nagel). Evaluation of the purity and concentration of the isolated genomic DNA samples was performed through gel electrophoresis on a 0.8% agarose gel and spectrophotometric analysis using Thermo Scientific's NANODROP 8000 spectrophotometer, respectively. Genotyping procedures were conducted employing the “Axiom_*Arachis*” single nucleotide polymorphism (SNP) array comprising 58,233 SNP markers, derived from re‐sequencing and RNA‐seq data of 41 groundnut accessions and wild diploid ancestors (Clevenger et al., 2017; Pandey et al., 2017). The mini‐core germplasm accessions DNA samples were genotyped on the Affymetrix GeneTitan platform following established protocols (Gangurde et al., 2020), and resultant genotypic data in CEL file format were archived. Subsequent SNP calling and data analysis were executed utilizing the Axiom Analysis Suite version 1.0 (Thermo Fisher Scientific) to enforce quality control (QC) measures, thereby selecting samples that successfully met the QC criteria for further analysis. Among the 58,233 SNPs obtained from the Axiom analysis suite, a subset of high‐quality SNPs meeting a minor allele frequency threshold of ≥0.05 and with a maximum allowable missing data per SNP fixed to <20% were filtered using Tassel v5.0 software. Consequently, 10,064 high‐quality SNPs were retained for subsequent association mapping studies. Visualization of the distribution and density of SNP markers across different chromosomes was achieved using the R package CMplot (https://github.com/YinLiLin/R‐CMplot).

### 2.4 Genome‐wide association study and identification of candidate genes

Best linear unbiased predictions were calculated for all 3 years for both the component traits PDI and PM separately. “Axiom_*Arachis*” array data with 58K SNPs were filtered and 10,064 polymorphic and high‐quality SNPs were selected. The GWAS was conducted using the above phenotypic and genotypic data files with six multi‐locus GWAS models of the “mrMLM” v4.0 package in R software. Models, namely, Multi‐locus Random‐SNP‐effect Mixed Linear Model (mrMLM), Fast multi‐locus random‐SNP‐effect Efficient Mixed Model Association (FASTmeEMMA), Fast multi‐locus random‐SNP‐effect Mixed Linear Model (FASTmrMLM), Polygenic‐background‐control‐based Least Angle Regression + Empirical Bayes (pLARmEB), Polygenic‐background‐control‐based Kruskal–Wallis test + Empirical Bayes (pKWmEB), and Iterative Screen and Select (ISIS) Expectation‐Maximization Bayesian LASSO (ISISEM‐BLASSO) were used for analysis. FASTmrMLM is the faster method with high statistical power with high accuracy in marker trait association (MTA) estimation in comparison to mrMLM (Tamba & Zhang, 2018). The only significant markers above −log_10_(*p* value) (Bonferroni correction) and above logarithm of the odds (LOD) score 3 were considered as significant markers. MTAs that were stable across at least 2 years (seasons) were considered as stable associations. Genes were identified from the closer region (100 kb upstream and downstream was considered) of significant associations using the “peanut base” database. The relation of these genes with the biotic stress resistance was checked in the literature and then the potential candidate genes for the stem rot resistance were finalized. Gene function was collected from the peanut base.

### 2.5 Gene ontology enrichment and pathway dissection

The gene ontology (GO) is a central resource for functional‐genomics research. Researchers rely on the functional annotations in the GO for hypothesis generation and couple it with high‐throughput biological data to enhance interpretation of results. GO analysis was done using the “Phytozome‐13” (https://phytozome‐next.jgi.doe.gov/) database, and we got the GO annotation for almost all the candidate genes that we got through this study. Bonferroni was used as threshold for significance of GO terms. List of genes was classified based on their association with the GO terms and grouped into different GO classes like biological processes (BPs), molecular function (MF), and cellular components (CCs). Pathway was dissected using “Phytozome 13” and “KEGG orthology” (KO) (https://www.genome.jp/kegg/ko.html) database. We derived KEGG (Kyoto Encyclopedia of Genes and Genomes) IDs and enzyme IDs from phytozome‐13 and they were used in the KO to derive the pathway involved in stem rot disease resistance.

### 2.6 Marker validation through allele mining and KASP assays

The study population consisted of 524 genotypes from four *Arachis* species (*A. hypogaea*, *A. duranensis*, *Arachis monticola*, and *Arachis cardenasii*), representing three growth habit types: Spanish, Valencia, and Virginia (Supporting Information File S1). This diverse population was used for allele mining, by genotyping all 524 genotypes for 13 identified loci using Axiom_*Arachis* array markers. Genotypes possessing all 13 favorable alleles were selected and subsequently subjected to phenotyping to confirm resistance, utilizing an oxalic acid assay protocol (Veerendrakumar et al., 2024).

### 2.7 Validation using KASP markers

Kompetitive allele‐specific PCR (KASP) assays targeting 13 MTAs were designed on the Intertek platform using 300‐bp flanking sequences (upstream and downstream) for each MTA. Validation was performed using a panel of 35 genotypes, comprising of landraces and breeding lines (intervarietal and interspecific) on the same platform.

## 3 RESULTS

### 3.1 Sick field phenotyping

Phenotyping of a groundnut minicore set in sick field trials revealed significant genetic variability (*p* < 0.01) for stem rot resistance. Analysis of variance showed no replication effects but highly significant genotype, temporal, and seasonal variations, highlighting genetic and environmental influences on disease progression. Both disease components (PDI and PM) showed full severity ranges (0%–100%), with PDI exhibiting higher heritability (63% vs. PM's 56%). Variance components indicated greater genetic than environmental contributions (genetic coefficient of variation: 66.07% PDI, 67.23% PM; environmental coefficient of variation: 50.19% PDI, 59% PM). Strong phenotypic correlations between PDI and PM were observed across years (*r* = 0.77–0.99) (Veerendrakumar et al., 2024).

### 3.2 Identification of associated genomic regions with stem rot resistance

**FIGURE 1:**
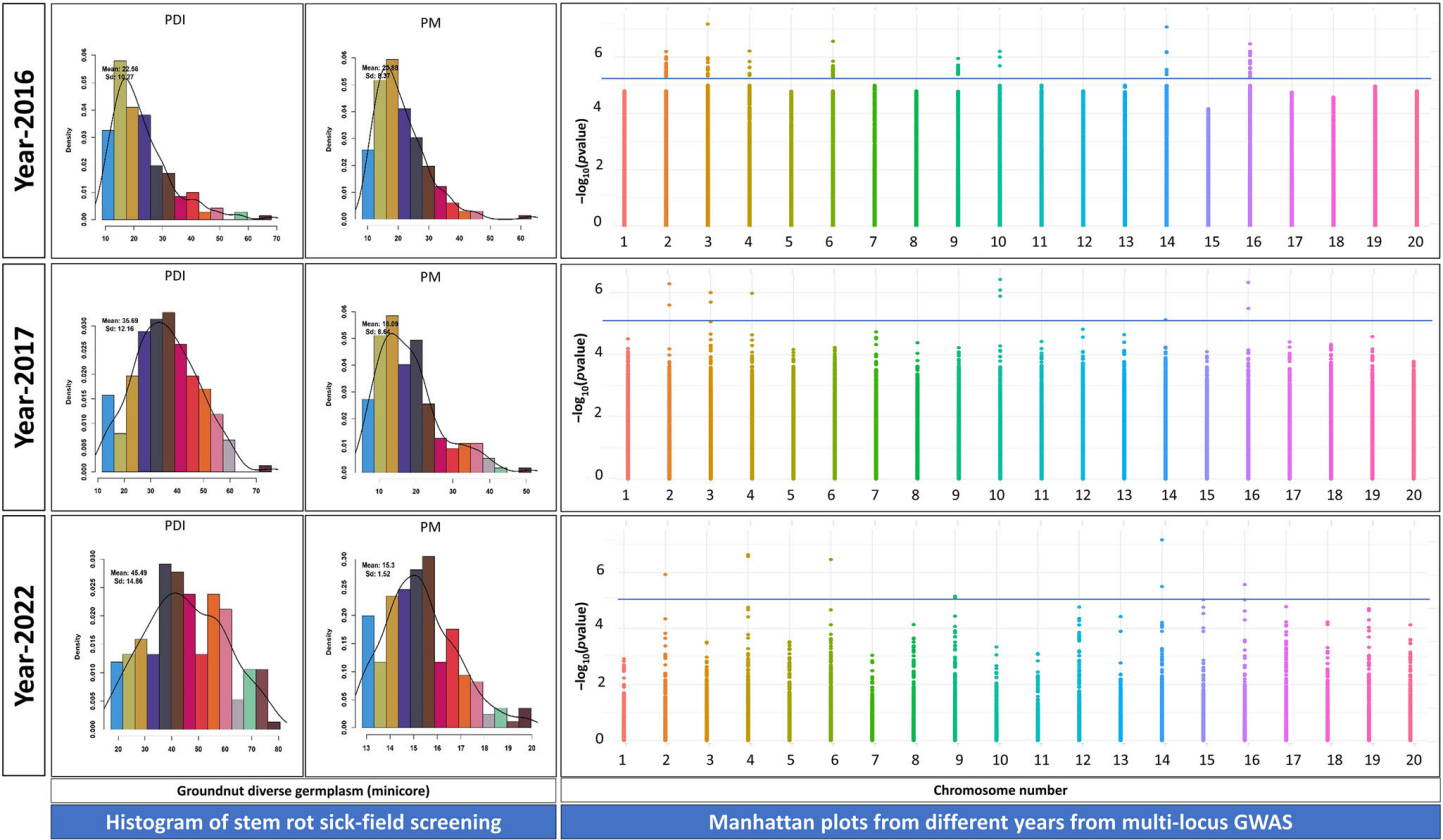
Histogram for showing the distribution of phenotypic data and Manhattan plots representing significant marker trait associations (MTAs) for the stem rot disease resistance during different years of screening. PDI, percent disease incidence; PM, percent mortality.

Association analysis was performed on 3 years of sick field evaluation data (SR_2016, SR_2017, and SR_2022) using the “Axiom_*Arachis*” array with 58K SNPs (Pandey et al., 2017) and the mrMLM package, which incorporates multi‐locus models (Veerendrakumar et al., 2024). Multi‐locus models are advantageous for GWAS as they eliminate the need for Bonferroni correction, thereby increasing the number of MTAs that can be identified. These models are noted for their high power and accuracy while maintaining a low false‐positive rate. The analysis identified 13 MTAs across eight chromosomes (Table 1), with LOD values ranging from 4.5 to 12.4 and *R* ^2^ values between 6.9% and 58%. GWAS conducted during rainy seasons identified distinct peaks across 3 years: eight peaks in 2016, six in 2017, and six in 2022 (Figure 1). Analysis using six statistical models revealed these peaks on chromosomes A02, A03, A04, A06, A09, A10, B04, and B06 (2016); A02, A03, A04, A10, B04, and B06 (2017); and A02, A04, A06, A09, B04, and B06 (2022). MTAs on chromosomes A02, A04, B04, and B06 were consistently detected in all 3 years. In contrast, MTAs on chromosomes A03, A06, A09, and A10 were identified in 2 of the 3 years.

**TABLE 1:** Marker trait associations for groundnut stem rot resistance identified from genome‐wide association study.

Sl. No.	Marker name	Chr	Position (bp)	LOD score	−log10(*p*)	*R* ^2^ (%)
1	AX_147213998	A02	67418614	8.46	9.36	36.05
2	AX_147216613	A03	31309264	8.23	9.13	23.00
3	AX_147217346	A03	110496307	5.91	6.74	10.89
4	AX_147219066	A04	2961855	10.34	11.28	47.40
5	AX_147219362	A04	8450510	5.88	6.71	6.95
6	AX_147226129	A06	102121026	11.66	12.64	58.58
7	AX_147225180	A06	18228746	4.55	5.32	16.15
8	AX_147234103	A09	111311680	5.45	6.26	46.96
9	AX_147234095	A09	111194838	11.44	12.41	44.74
10	AX_147237286	A10	108724599	11.86	12.84	21.78
11	AX_147247823	B04	98007031	12.49	13.48	30.74
12	AX_147247654	B04	66038907	4.57	5.34	17.05
13	AX_147251504	B06	115522	4.83	5.61	44.25

Abbreviations: Chr, chromosome; LOD, logarithm of the odds.

### 3.3 Identification of allele combinations associated with stem rot resistance

**FIGURE 2:**
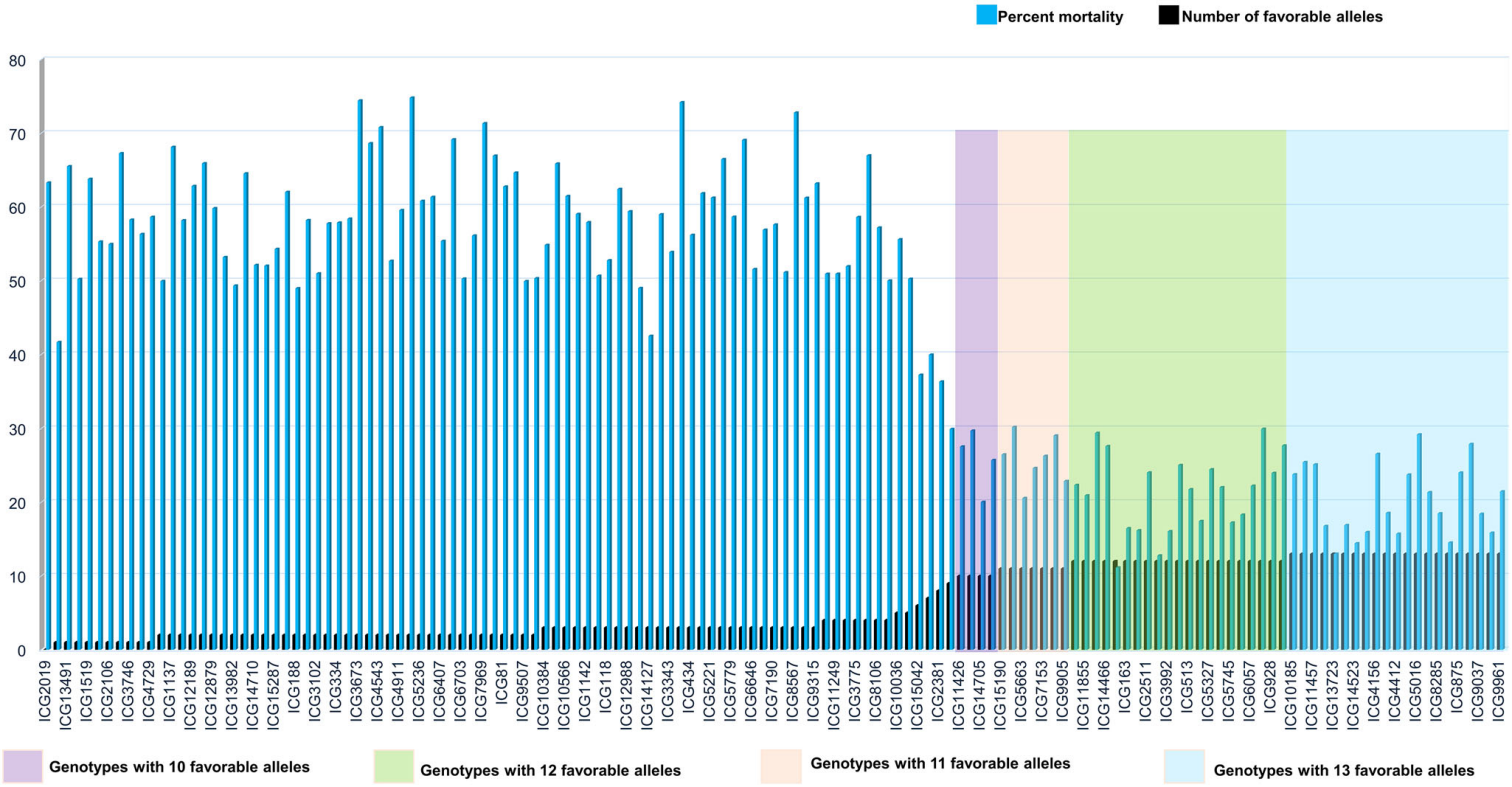
Reaction of minicore to stem rot disease with increase in favorable alleles. An increase in the number of favorable alleles increased the amount of resistance offered by the genotype. A drastic increase in resistance was observed with an increase in favorable alleles from 5 to10, and after 10, the amount of resistance was almost similar. This shows single specific segment (gene) cannot offer resistance to stem rot disease, and it requires these 13 favorable alleles to provide stable resistance. Different color highlights are provided for 10, 11, 12, 13 favorable alleles.

**FIGURE 3:**
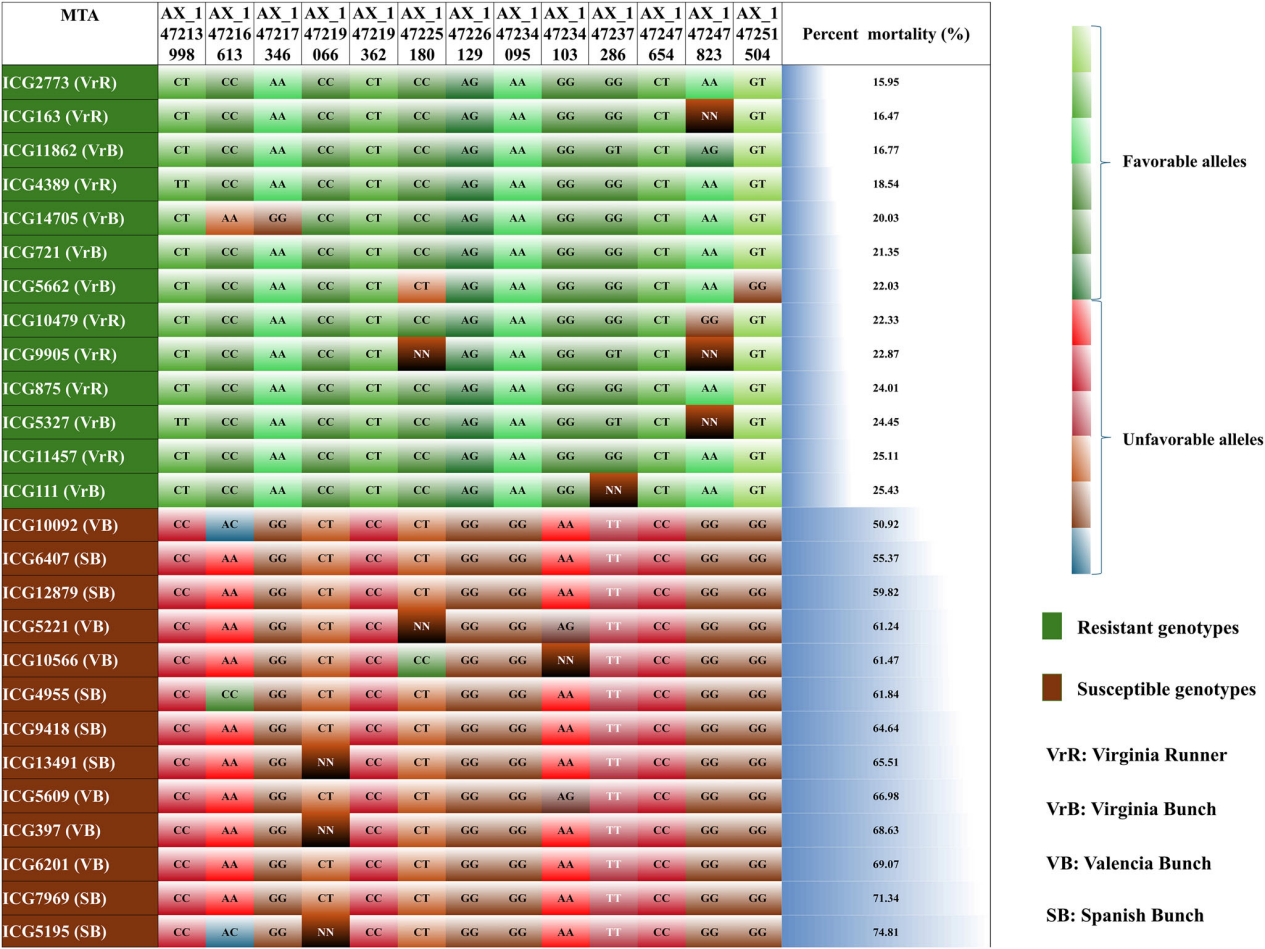
Representation of allele combinations required by genotype to show resistance to stem rot disease. Genotypes showing the extreme phenotype for the percent mortality were compared for the allelic differences for the identified 13 significant single nucleotide polymorphisms (SNPs), showing that all the resistant genotypes showed favorable alleles and susceptible genotypes showed unfavorable alleles. It was also found that all the resistance genotypes belong to Virginia type groundnut. MTA, marker trait association.

To elucidate the impact of allelic variation on stem rot resistance, 13 significant SNPs (MTAs) located across eight chromosomes associated with resistance were selected. These associations, with −log10 *p* values ranging from 5.32 to 13.48, indicate a high level of statistical significance. To evaluate the resistance conferred by these loci, a panel of extreme phenotypes was assembled. Results showed that each locus cumulatively enhances resistance: as the number of loci with favorable alleles increases, the resistance level in the genotype also increases (Figure 2). This specific combination of favorable alleles directly influences resistance, as evidenced by a reduced PM in genotypes carrying these alleles. The allele combination across these 13 loci represents a comprehensive genomic profile for stem rot resistance (Figure 3). This knowledge is invaluable for breeding programs, providing a robust approach for managing stem rot disease through host–plant resistance.

### 3.4 Candidate gene discovery for stem rot resistance

Candidate genes within a 100 kb region upstream and downstream of each MTA were extracted using the PeanutBase database. The analysis identified several key genes that play a vital role in disease resistance, specifically aiding cells in defense, tolerance, and recovery from oxalic acid exposure. Many genes with functionally similar copies were found across various MTAs (Table 2), underscoring their significance in resistance mechanisms. A total of 145 genes directly related to biotic stress resistance were identified within this 100 kb region of the 13 MTAs (listed in the gene list in the Supporting Information), indicating the polygenic inheritance of stem rot resistance. Stem rot resistance requires a precise combination of alleles, as environmental factors can influence gene expression; however, the presence of these alleles consistently limits mortality to below 30%. As part of the defense response, fungal pathogens are recognized by plant receptor proteins and resistant genes (*R* genes). These responses were categorized by function, with receptor‐like kinases (RLKs) as the primary group involved in fungal recognition. RLK proteins included protein kinases, wall‐associated receptor kinases, serine/threonine protein kinases, lucine‐rich repeat (LRR) receptors, and EGF‐like calcium‐binding domains, among others. Ten receptors were distributed across four chromosomes, aligning with findings from a previous RNA‐seq study (Bosamia et al., 2020), which identified similar compounds (Table 3). *R* genes related to specific functions include NBS‐LRR proteins, LRR and NB‐ARC domain disease‐resistant proteins, leucine‐rich repeats, peroxidase superfamily proteins, and a range of others such as chitinase A, cytochrome P450, and glycosyl hydrolase proteins. Over 25 distinct *R* genes were located in close proximity to MTAs, further supported by RNA‐seq data (Bosamia et al., 2020). Transcription factors (TFs) influencing these defense responses included MADS‐box, CSL zinc fingers, MYM‐type zinc fingers, GRF zinc finger, and CCHC‐type zinc finger TFs. The zinc finger class was notably abundant, followed by RING/FYVE/PHD and GRF classes. These TFs largely matched transcripts reported in RNA‐seq experiments, supporting the consistency of these GWAS findings with previous transcriptomic study (Bosamia et al., 2020).

**TABLE 2:** Genes found (with respect to gene function) in the upstream and downstream region of the marker trait associations (MTAs) with their respective copy number.

Sl. No.	Gene function	Chromosome	Number
1	Protein kinase family protein	4, 9, 16	10
2	Leucine‐rich repeat (LRR)	3, 4, 9	8
3	Wall‐associated receptor kinase	4	8
4	Cytochrome P450 superfamily protein	2, 4, 9	5
5	Glycoside hydrolase	6, 9	4
6	Peroxidase activity	4	4
7	Serine/threonine‐protein phosphatase 7	3, 9	4
8	Zinc finger protein	6, 9	4
9	Folate/biopterin transporter	9	3
10	GRF zinc finger protein	3, 9	3
11	Lactoylglutathione lyase	4	3
12	Pyridoxal phosphate (PLP)‐dependent transferases superfamily protein	9	3
13	RING/FYVE/PHD zinc finger superfamily protein	9, 14	3
14	Trihelix transcription factor	9	3
15	Ascorbate oxidase	6, 16	2
16	Calcium dependent protein kinase	2, 16	2
17	Cellulose synthase	2	2
18	Copper amine oxidase family protein	9	2
19	DEAD‐box ATP‐dependent RNA helicase	16	2
20	Histone‐lysine N‐methyltransferase	6	2
21	Large proline‐rich protein BAG6	6, 16	2
22	MADS‐box transcription factor 20	2, 9	2
23	Magnesium transporter MRS2/LPE10	9	2
24	Pentatricopeptide repeat (PPR) superfamily protein	14, 16	2
25	Peptidase S9A, prolyl oligopeptidase	6, 16	2
26	Genes with low copy number		1

**TABLE 3:** Common genes identified in our genome‐wide association study (GWAS) analysis and RNA sequencing (RNA‐seq) study by Bosamia et al. (2020).

Gene ID	Gene description	Organism	Chromosome	Start	End
*Aradu.1661Y*	CCHC‐type zinc finger	*A. duranensis*	Aradu.A09	117245635	117248243
*Aradu.4C43R*	MYM‐type zinc fingers	*A. duranensis*	Aradu.A04	8445965	8446930
*Aradu.50IFA*	Octicosapeptide/Phox/Bem1p family protein	*A. duranensis*	Aradu.A04	2969578	2970781
*Aradu.7AG2S*	S‐adenosyl‐L‐methionine‐dependent methyltransferase	*A. duranensis*	Aradu.A04	9858629	9864724
*Aradu.7ZK0R*	Peroxidase superfamily proteins	*A. duranensis*	Aradu.A04	8360526	8364217
*Aradu.80LAN*	Lucine‐rich repeats	*A. duranensis*	Aradu.A03	110428984	110430861
*Aradu.A9Z84*	Trihelix transcription factors GT‐2	*A. duranensis*	Aradu.A09	117233531	117234625
*Aradu.CRB2F*	LRR receptors	*A. duranensis*	Aradu.A09	116939166	116942708
*Aradu.G04HZ*	RING/FYVE/PHD zing finger	*A. duranensis*	Aradu.A09	111321581	111327778
*Aradu.HWT05*	Serine/threonine protein kinase	*A. duranensis*	Aradu.A03	110502346	110505487
*Aradu.IF9NE*	Glycosyl hydrolase	*A. duranensis*	Aradu.A09	112356174	112356765
*Aradu.KR1YF*	Polyketide cyclase/hydrase protein	*A. duranensis*	Aradu.A02	91664080	91668585
*Aradu.L1NQG*	EGF like calcium binding domain	*A. duranensis*	Aradu.A09	112375767	112379859
*Aradu.LF7PI*	Homocysteine S‐methyltransferase	*A. duranensis*	Aradu.A09	111136459	111138608
*Aradu.SE2EU*	Cytochrome P450 superfamily protein	*A. duranensis*	Aradu.A04	9877895	9881826
*Aradu.SGG9Q*	MADS‐box TF	*A. duranensis*	Aradu.A02	91676432	91681325
*Aradu.T4YKC*	CSL zinc fingers	*A. duranensis*	Aradu.A02	91729006	91731267
*Aradu.VDD1W*	Wall associated receptor kinases	*A. duranensis*	Aradu.A04	9833296	9843589
*Aradu.W5C9S*	Chitinase A	*A. duranensis*	Aradu.A09	116929900	116931246
*Aradu.WH0UE*	LRR and NB‐ARC domain disease resistant proteins	*A. duranensis*	Aradu.A02	91571749	91590358
*Aradu.XBA07*	Cysteine‐type peptidase activity	*A. duranensis*	Aradu.A02	67409494	67410805
*Aradu.YUF6V*	NBS‐LRR proteins	*A. duranensis*	Aradu.A04	9866582	9869557
*Aradu.Z5PZW*	GRF zinc finger	*A. duranensis*	Aradu.A09	111260289	111261409
*Araip.6C47W*	Dead‐box protein	*A. ipaensis*	Araip.B06	154379	161658
*Araip.K2I95*	Tetracopeptide repeat (TPR) superfamily protein	*A. ipaensis*	Araip.B04	10790632	10793265
*Araip.XI1AM*	Syringolide‐induced protein	*A. ipaensis*	Araip.B06	197785	198651

Abbreviations: LRR, lucine‐rich repeats; TF, transcription factor.

### 3.5 Enrichment analysis

**FIGURE 4:**
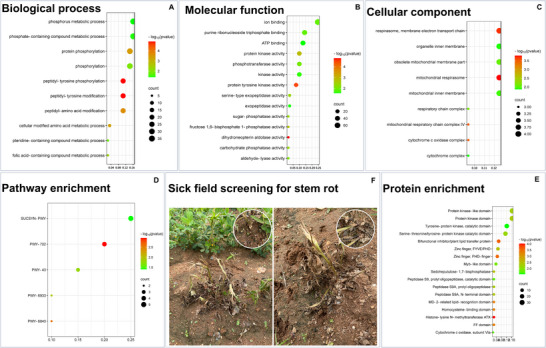
Gene ontology enrichment (A, B, C), pathway (D), and protein enrichment (E) for the genes that are found through genome‐wide association study (GWAS) for stem rot resistance along with the representation of comparative reaction of stem rot resistant and susceptible lines (F). Enrichment analysis showing the genes in significant correlation with gene ontology (GO) terms into biological processes, molecular function, and cellular components. Stem rot screening showing the intensity of disease at the time of sick field screening (F).

Ontologies provide a structured vocabulary to describe domain knowledge, enabling systematic categorization of biological data. GO annotations specify the roles of an organism's genes in BPs, cellular localization, and MF. For this study, diploid gene IDs were converted to tetraploid gene IDs and subjected to GO analysis, involving 237 *Arachis hypogea* gene IDs, of which 220 were GO‐annotated. These GO annotations provide valuable insights into gene functions, with each annotation supported by evidence codes that specify the basis for the association between genes and GO terms. GO enrichment analysis was conducted for 220 genes associated with stem rot resistance, with differentially expressed genes (DEGs) between susceptible and resistant genotypes categorized into BPs, CCs, and MFs. Among the top nine upregulated DEGs, peptidyl‐tyrosine phosphorylation (GO:0018108) emerged as the most significantly upregulated BP in resistant genotypes (Figure 4). Other notable processes in resistant genotypes included cellular modified amino acid metabolic process (GO:0006575), folic acid‐containing compound metabolic process (GO:0006760), and peptidyl‐amino acid modification (GO:0018193). For CCs, the most significantly upregulated elements included mitochondrial respirasome (GO:0005746), cytochrome c oxidase complex (GO:0045277), organelle inner membrane (GO:0019866), and mitochondrial membrane part (GO:0044455). In MFs, the upregulated terms included dihydroneopterin aldolase activity (GO:0004150), protein tyrosine kinase activity (GO:0004713), serine‐type exopeptidase activity (GO:0070008), aldehyde‐lyase activity (GO:0016832), and exopeptidase activity (GO:0008238). Protein enrichment analysis highlighted protein groups enhanced by the candidate genes, notably those with protein kinase domain, tyrosine‐protein kinase, serine‐threonine kinase, and zinc finger FYVE/PHD (Figure 4), which are significant for stem rot resistance. The infection response also included antioxidants and detoxifying agents, with relevant genes identified in this study, such as those encoding cytochrome P450 superfamily proteins, peroxidase superfamily proteins, and L‐ascorbate oxidase homologs.

### 3.6 Uncovered pathway associated with stem rot resistance in groundnut

**FIGURE 5:**
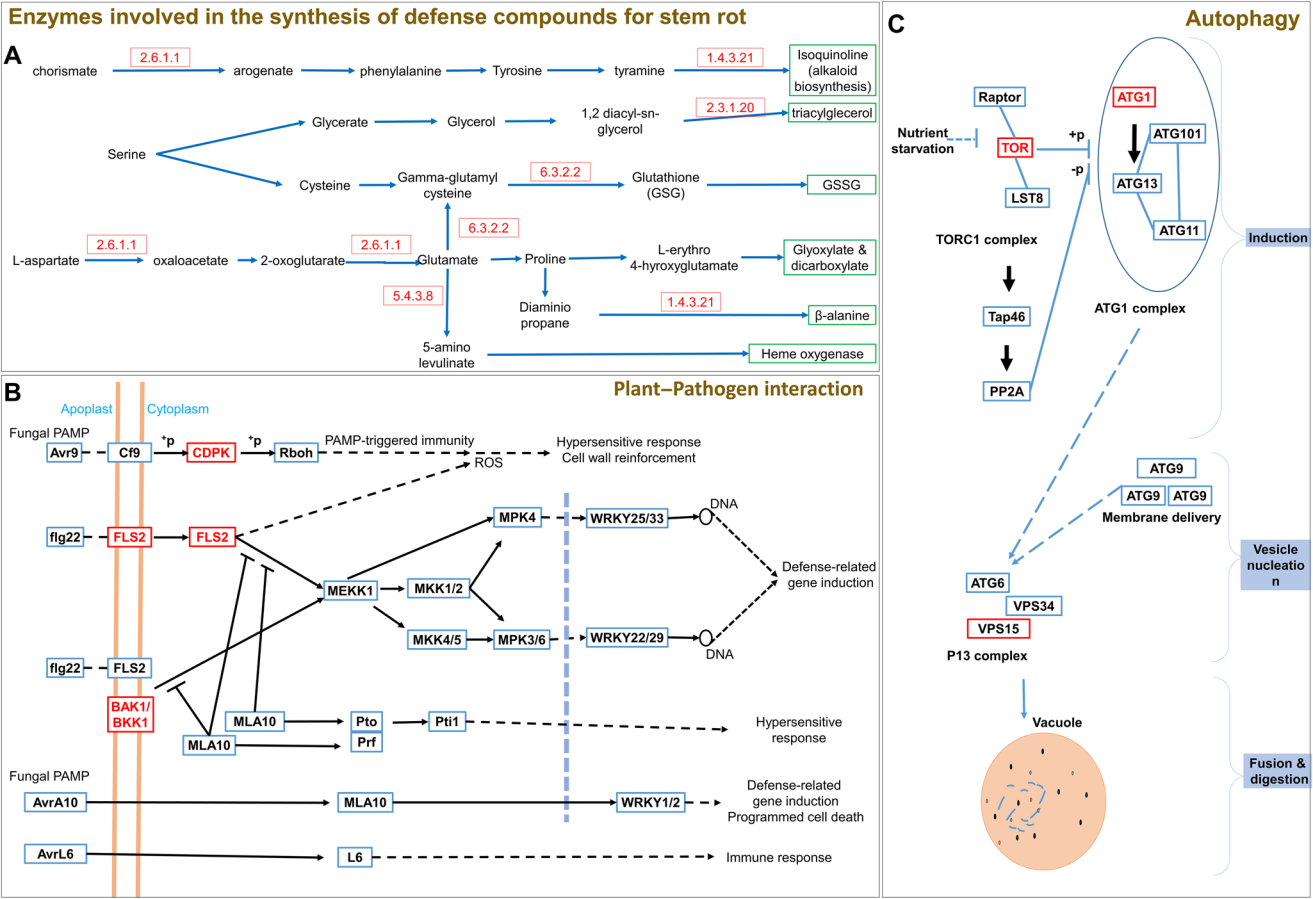
Pathway representing the involvement of identified candidate genes in the formation of different molecules providing the potential for a plant to withstand stem rot disease. All the genes/enzymes mentioned in red boxes are the genes found in this study. The compounds produced by the plant to defend and protect the cell from pathogen and their toxic chemicals (A), plant–pathogen interaction and different receptor‐like kinases (RLKs) produced and different responses by the plant (B), and the final decision taken by the plant if it is unable to protect the cell during severe infection, that is, autophagy (C).

Individual gene analysis offers insight into specific genes but does not capture the entire resistance mechanism. For a comprehensive understanding, dissecting the pathway involved in stem rot resistance is essential. This study outlines the pathway by which resistance developed in the plant against stem rot disease (Figure 5). As the pathogen invades plant tissue, it secretes high levels of oxalic acid, which degrades the plant cell wall. In response, the plant initiates defense mechanisms, producing compounds to counteract the pathogen and neutralize the oxalic acid. During this plant–pathogen interaction, the FLS2 gene (*arahy.Tifrunner.gnm1.ann1.D2QNFF*) becomes active, producing defense‐related proteins. Based on the cell's condition, the plant then assesses whether to rescue the cell or trigger cell death to protect neighboring cells. If a cell is beyond repair, the BAK1/BKK1 gene (*arahy.Tifrunner.gnm1.ann1.DXLT9X*) activates, initiating PAMP‐triggered immunity with the aid of the CDPK gene (*arahy.Tifrunner.gnm1.ann1.M8Q52W*), leading to programmed cell death. The process of programmed cell death is further supported by autophagy‐related genes such as TOR and ATG1, which are essential for autophagy initiation, while VPS15 is involved in vesicle nucleation, leading to fusion and digestion. On the other hand, if a cell is viable, the plant activates additional defenses. It synthesizes isoquinoline, an antifungal compound, with the assistance of enzymes aspartate transaminase (2.6.1.1) (*arahy.Tifrunner.gnm1.ann1.6D6Z2R*) and primary‐amine oxidase (1.4.3.21) (*arahy.Tifrunner.gnm1.ann1.JV3LD5*). Additional biochemical responses include the production of triglycerides by the enzyme diacylglycerol O‐acyltransferase (2.3.1.20) (*arahy.Tifrunner.gnm1.ann1.WVQY2R*), which mitigates biotic stress, and glutathione disulfide (GSSG) by glutamate–cysteine ligase (6.3.2.2) (*arahy.Tifrunner.gnm1.ann1.Y73929*), which maintains cellular homeostasis. Detoxification during stress is further supported by glyoxylate and dicarboxylate, synthesized via aspartate transaminase (*arahy.Tifrunner.gnm1.ann1.6D6Z2R*) and glutamate–cysteine ligase (*arahy.Tifrunner.gnm1.ann1.Y73929*). β‐alanine, produced by primary‐amine oxidase (*arahy.Tifrunner.gnm1.ann1.JV3LD5*), serves as a defense compound against biotic stresses, while heme oxygenase, generated by glutamate‐1‐semialdehyde 2,1‐aminomutase (5.4.3.8) (*arahy.Tifrunner.gnm1.ann1.M8Q52W*), exerts antioxidative and cytoprotective effects to support the plant under stress.

### 3.7 Marker validation through allele mining and phenotype confirmation

**FIGURE 6:**
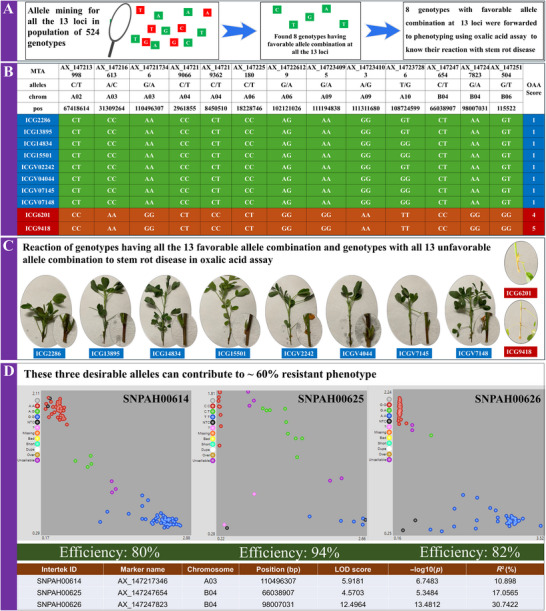
Allele mining and validation of identified single nucleotide polymorphisms (SNPs) for stem rot resistance. (A) Allele mining for favorable alleles in a population of 524 genotypes, (B) genotypes selected having favorable alleles from allele mining forwarded for phenotypic confirmation, (C) phenotyping resulted in showing very low lesion/no lesion on the stem on treatment with oxalic acid assay, and (D) second stage validation using kompetitive allele‐specific PCR (KASP) assays showing finalized three markers for marker‐assisted selection for stem rot resistance. LOD, logarithm of the odds.

To validate the findings, allele mining was performed on a population of 524 genotypes to identify those possessing the complete combination of favorable alleles across the 13 identified loci. The Axiom_*Arachis* array assay SNP markers facilitated this allele mining process. Among the genotypes examined, eight (ICG 2286, ICG 13895, ICG 14834, ICG 15501, ICGV 02242, ICGV 04044, ICGV 07145, and ICGV 07148) were found to carry all favorable alleles at the 13 loci identified through GWAS (Figure 6). This group included four landraces and four breeding lines, which were subsequently subjected to phenotyping via an oxalic acid assay to confirm resistance. Notably, each genotype with all 13 favorable alleles exhibited a very low wilting score of 1 (Figure 6), indicating high resistance to stem rot. These results confirm that stem rot resistance is a polygenic trait, controlled by the cumulative effects of 13 genomic regions, all of which are essential for robust resistance. As the number of favorable alleles decreases, resistance levels also decline proportionally. This study validates these 13 SNP markers identified through GWAS, reaffirming their strong association with stem rot resistance.

### 3.8 Validation using KASP assays

KASP assays were developed for 13 SNPs, three of which effectively distinguished resistant and susceptible lines. These markers—SnpAH00614 (located on gene ***AhSR001***, encoding an auxin‐related protein 2), SnpAH00625 (on ***AhSR002***, a histidine triad protein gene), and SnpAH00626 (on ***AhSR003***, an E3 ubiquitin‐protein ligase DRIP2 gene)—showed differentiation efficiencies of 80%, 94%, and 82%, respectively. The validated markers are accessible on the Intertek genotyping platform and can be utilized for marker‐assisted selection (MAS) of resistant genotypes in future breeding programs.

## 4 DISCUSSION

Stem rot, caused by *S. rolfsii*, is a significant economic threat to groundnut production in many Asian and sub‐Saharan countries, particularly within resource‐limited environments. While agronomic (Spadaro & Gullino, 2005) and chemical (Grichar, 1995) methods have been explored for disease management, their success has been limited. Utilizing host plant resistance offers a safer and cost‐effective alternative for disease management. Identifying resistant genotypes is critical for understanding the mechanisms behind resistance and for developing resistant varieties. For the precise introgression of resistance traits into elite germplasm, markers are needed to confirm the presence of the genomic regions associated with resistance. Only a few mapping studies have targeted the QTLs for stem rot resistance (Agmon et al., 2022; Bera et al., 2016; Bosamia et al., 2020; Cui et al., 2020; Dodia et al., 2019; Luo et al., 2020), with one GWAS reported on Chinese minicore (Yan et al., 2023). Although the trait shows high heritability of phenotype, translating this into high PVE by detected QTLs has been challenging, likely due to the influence of numerous small‐effect loci contributing to this resistance trait (Luo et al., 2020).

Reported studies remain insufficient for developing a stem rot‐resistant groundnut variety, underscoring the need for further investigation. The present study aimed to identify key genomic regions and pathways associated with stem rot resistance using a mini core germplasm of 184 accessions. Multi‐locus GWAS models were employed to improve accuracy by reducing false positives in comparison to single‐locus models and identifying regions with both interaction effects and small‐effect loci (Vikas et al., 2022). GWAS results identified 13 MTAs across eight chromosomes, suggesting that stem rot resistance is polygenic, requiring the cumulative action of multiple genes to achieve high resistance to pathogen infection. These MTAs were located on chromosomes A02, A03, A04, A06, A09, B04, and B06, aligning with previous findings: A03 was reported by three studies (Dodia et al., 2019; Luo et al., 2020; Yan et al., 2023), A04 by three studies (Bosamia et al., 2020; Luo et al., 2020; Yan et al., 2023), A06 by two studies (Bosamia et al., 2020; Luo et al., 2020), B04 by three studies (Dodia et al., 2019; Luo et al., 2020; Yan et al., 2023), and B06 by two studies (Dodia et al., 2019; Luo et al., 2020). The high *R* ^2^ values of each MTA indicate substantial contributions to phenotypic variance, marking these regions as priorities for breeding resistant genotypes. High LOD scores confirm the MTAs’ significant influence on resistance traits, clearly differentiating resistant from susceptible genotypes. An increased number of favorable alleles among these 13 loci proportionally enhances resistance to *S. rolfsii* (Figure 2), with individual and interactive effects contributing to stem rot resistance. These loci are thus critical targets in breeding programs focused on developing stem rot‐resistant varieties. Notably, all resistant genotypes identified in this study belong to the Virginia type, with no other types demonstrating resistance to stem rot. This suggests that future resistance breeding efforts should concentrate on Virginia types, although resistance levels within Virginia genotypes vary. Only those with this specific favorable allele combination at the 13 loci exhibit resistance, indicating the necessity of these loci for effective stem rot resistance.

Genes located near MTAs were identified and verified through literature, revealing 145 genes related to biotic stress resistance. Many of these genes include multiple functional copies, with key genes implicated in this resistance trait such as wall‐associated receptor kinases, chitinase proteins, disease‐resistance proteins, and peroxidase superfamily proteins. Similar major genes were previously reported in QTL mapping (Luo et al., 2020) and a GWAS study (Yan et al., 2023). Additional significant genes identified in this study include those with roles in protein kinase function, calcium‐dependent protein kinases (calcium‐binding domain), thioredoxin 3, and zinc finger superfamily proteins, which have also been reported in a Chinese minicore GWAS study (Yan et al., 2023). Other genes associated with stem rot resistance include NBS‐LRR, folate transporter, copper amine oxidase family protein, tetratricopeptide repeat, DEAD‐box ATP‐dependent RNA helicase, phytosulfokines, and serine endopeptidase activity. Nearly all functions of these genes, such as RLKs, *R* genes, and TFs, align with the transcripts identified in a stem rot RNA‐seq study (Bosamia et al., 2020), showing a strong correlation between genomic data and transcriptional responses in resistant genotypes. The genes identified in this study encompass essential roles across the plant–pathogen interaction stages: pathogen recognition (via RLKs), interaction with pathogen‐derived substances, antioxidant response, detoxification, and *R* gene‐mediated protection against *S. rolfsii*. This comprehensive gene capture suggests that the genetic basis for stem rot resistance in groundnut is well‐characterized, covering all necessary stages for a robust defensive response to pathogen infection.

After confirming the relation of these genes with stem rot resistance, we also performed GO enrichment analysis. This divides the DEGs into GO terms such as BPs, MF, and CCs. GO enrichment analysis assigned statistical significance to each GO term associated with the GWAS‐identified gene list. We identified many GO terms responsible for the stem rot resistance under BP, MF, CC, which will help us know the involvement of these genes in different functions and parts of the plant cell. This will help us in moving ahead with pathway dissection. Pathway of stem rot had three parts. First, the plant identifies the stress due to infection of pathogen and the plant–pathogen interaction begins, as we all know the primary product produced by pathogen is oxalic acid and it acts on calcium ions of the plant cell wall, beginning the degradation of the cell wall. Finally, based on the infection levels, plant initiates its defense mechanisms, like PAMP‐triggered immunity, hypersensitive response, defense‐related gene induction, and reactive oxygen species production, which then leads to autophagy or programmed cell death in susceptible plants. Resistant plants will have the *R* genes and RLKs and TFs for production of required compounds (compounds mentioned in Figure 5) to overcome the infection and save/recover the cell/tissue and keep the plant healthy. This is the main pathway involved in stem rot resistance and this resistance is governed by the cumulative gene action of all the genes present in those reported loci in this study. This combination of alleles can make a plant resistant and keep the PM level below 30% (from the sick‐field data of 3 years).

## 5 CONCLUSIONS

Stem rot disease can cause yield losses up to 80% in groundnut and host plant resistance is the way forward to address this difficult‐to‐manage disease. GWAS on ICRISAT minicore has revealed significantly associated genomic regions with stem rot resistance on eight different chromosomes. The vital genes associated with the stem rot resistance have been identified and are highly correlated with a study done through RNA‐seq. This shows the high relatedness of these studies at gene and transcript level. Evaluation was done for genotypes belonging to Spanish, Valencia, and Virginia‐type growth habits. It has been confirmed that the resistance offered to this disease is only from Virginia type groundnut. We have also confirmed that RLKs play a pivotal role in perceiving *S. rolfsii* infection and the *R* genes, such as NBS‐LRR, NB‐ARC, peroxidases, and so on, helping the plant to resist the pathogen and sustain the stress. Pathway dissection has shown that the enzymes produced by the genes identified in this study are producing the compounds helping the plant to resist and recover the damaged parts of the cell. Finally, three genes, *AhSR001*, *AhSR002*, *AhSR003*, identified in this study have been validated using KASP assays and confirmed their association with stem rot disease resistance. We have narrowed it down to three KASP markers that can provide nearly 60% resistant phenotype, but for higher resistance, all 13 favorable alleles must be present. These validated markers can be used in breeding programs for the development of resistant varieties through MAS and marker‐assisted backcross breeding.

## AUTHOR CONTRIBUTIONS

**H. V. Veerendrakumar**: Conceptualization; data curation; formal analysis; investigation; methodology; software; validation; visualization; writing—original draft. **Hari Kishan Sudini**: Conceptualization; funding acquisition; methodology; resources; supervision; writing—review and editing. **Bangaru Kiranmayee**: Investigation; software; writing—review and editing. **Talwar Devika**: Data curation; software; visualization. **Sunil S. Gangurde**: Conceptualization; software; visualization. **R. P. Vasanthi**: Conceptualization; supervision; writing—review and editing. **A. R. Nirmal Kumar**: Conceptualization; formal analysis; writing—review and editing. **Sandip K. Bera**: Formal analysis; visualization; writing—review and editing. **Baozhu Guo**: Data curation; software; writing—review and editing. **Boshou Liao**: Formal analysis; writing—review and editing. **Rajeev K. Varshney**: Data curation; formal analysis; writing—review and editing. **Manish K. Pandey**: Conceptualization; funding acquisition; project administration; resources; supervision; writing—review and editing.

## CONFLICT OF INTEREST STATEMENT

The authors declare no conflicts of interest.

## Supporting information



Includes the information on biotic stress related genes identified through GWAS (Sheet 1). KEGG IDs and Enzyme ID of identified genes derived using Phytozome 13 database (Sheet 2). Sequence of markers used in Axiom_*Arachis* array for validation (sheet 3). List of genotypes used for validation (Sheet 4).

## Data Availability

The datasets generated in the study are either presented in the supplementary information associated with the article or can be provided on request to the corresponding author.

## References

[tpg270089-bib-0001] Agmon, S. , Kunta, S. , Yelin, M. D. , Moy, J. , Ibdah, M. , Harel, A. , Rabinovitch, O. , Levy, Y. , & Hovav, R. (2022). Mapping of stem rot resistance in peanut indicates significant effect for plant architecture locus. Crop Science, 62(6), 2197–2211. 10.1002/CSC2.20803

[tpg270089-bib-0002] Bera, S. K. , Kamdar, J. H. , Kasundra, S. V. , & Ajay, B. C. (2016). A novel QTL governing resistance to stem rot disease caused by *Sclerotium rolfsii* in peanut. Australasian Plant Pathology, 45(6), 637–644. 10.1007/S13313-016-0448-X

[tpg270089-bib-0003] Bertioli, D. J. , Cannon, S. B. , Froenicke, L. , Huang, G. , Farmer, A. D. , Cannon, E. K. S. , Liu, X. , Gao, D. , Clevenger, J. , Dash, S. , Ren, L. , Moretzsohn, M. C. , Shirasawa, K. , Huang, W. , Vidigal, B. , Abernathy, B. , Chu, Y. , Niederhuth, C. E. , Umale, P. , & Ozias‐Akins, P. (2016). The genome sequences of *Arachis duranensis* and *Arachis ipaensis*, the diploid ancestors of cultivated peanut. Nature Genetics, 48(4), 438–446. 10.1038/ng.3517 26901068

[tpg270089-bib-0004] Bosamia, T. C. , Dodi, S. M. , Mishr, G. P. , Ahmad, S. , Joshi, B. , Thirumalaisam, P. P. , Kumar, N. , Rathnakuma, A. L. , Sangh, C. , Kumar, A. , & Thankappan, R. (2020). Unraveling the mechanisms of resistance to *Sclerotium rolfsii* in peanut (*Arachis hypogaea* L.) using comparative RNA‐Seq analysis of resistant and susceptible genotypes. PLoS One, 15(8), e0236823. 10.1371/JOURNAL.PONE.0236823 32745143 PMC7398544

[tpg270089-bib-0005] Clevenger, J. , Chu, Y. , Chavarro, C. , Hovav, R. , Burow, M. , Nayak, S. N. , Chitikineni, A. , Isleib, T. G. , Holbrook, C. C. , Jackson, S. A. , Varshney, R. K. , & Ozias‐Akins, P. (2017). Genome‐wide SNP genotyping resolves signatures of selection and tetrasomic recombination in peanut. Molecular Plant, 10, 309–322. 10.1016/j.molp.2016.11.015 27993622 PMC5315502

[tpg270089-bib-0006] Cui, R. , Clevenger, J. , Chu, Y. , Brenneman, T. , Isleib, T. G. , Holbrook, C. C. , & Ozias‐Akins, P. (2020). Quantitative trait loci sequencing–derived molecular markers for selection of stem rot resistance in peanut. Crop Science, 60(4), 2008–2018. 10.1002/CSC2.20047

[tpg270089-bib-0008] Dodia, S. M. , Joshi, B. , Gangurde, S. S. , Thirumalaisamy, P. P. , Mishra, G. P. , Narandrakumar, D. , Soni, P. , Rathnakumar, A. L. , Dobaria, J. R. , Sangh, C. , Chitikineni, A. , Chanda, S. V. , Pandey, M. K. , Varshney, R. K. , & Thankappan, R. (2019). Genotyping‐by‐sequencing based genetic mapping reveals large number of epistatic interactions for stem rot resistance in groundnut. Theoretical and Applied Genetics, 132(4), 1001–1016. 10.1007/S00122-018-3255-7 30539317

[tpg270089-bib-0023] FAO . (2023). *FAOSTAT: Crops and livestock products*. Food and Agriculture Organization of the United Nations. https://www.fao.org/faostat/

[tpg270089-bib-0009] Gangurde, S. S. , Wang, H. , Yaduru, S. , Pandey, M. K. , Fountain, J. C. , Chu, Y. , Isleib, T. , Holbrook, C. C. , Xavier, A. , Culbreath, A. K. , Ozias‐Akins, P. , Varshney, R. K. , & Guo, B. (2020). Nested‐association mapping (NAM)‐based genetic dissection uncovers candidate genes for seed and pod weights in peanut (*Arachis hypogaea*). Plant Biotechnology Journal, 18(6), 1457–1471. 10.1111/PBI.13311 31808273 PMC7206994

[tpg270089-bib-0010] Grichar, W. J. (1995). Management of stem rot of peanuts (*Arachis hypogaea*) caused by *Sclerotium rolfsii* with fungicides. Crop Protection, 14(2), 111–115. 10.1016/0261-2194(95)92864-J

[tpg270089-bib-0011] Jadon, K. S. , Thirumalaisamy, P. P. , Kumar, V. , Koradia, V. G. , & Padavi, R. D. (2015). Management of soil borne diseases of groundnut through seed dressing fungicides. Crop Protection, 78, 198–203. 10.1016/J.CROPRO.2015.08.021

[tpg270089-bib-0012] Kokalis‐Burelle, N. , Mahaffee, W. F. , Rodríguez‐Kábana, R. , Kloepper, J. W. , & Bowen, K. L. (2002). Effects of switchgrass (*Panicum virgatum*) rotations with peanut (*Arachis hypogaea* L.) on nematode populations and soil microflora. Journal of Nematology, 34(2), 98–105. 19265915 PMC2620544

[tpg270089-bib-0013] Luo, Z. , Cui, R. , Chavarro, C. , Tseng, Y. C. , Zhou, H. , Peng, Z. , Chu, Y. , Yang, X. , Lopez, Y. , Tillman, B. , Dufault, N. , Brenneman, T. , Isleib, T. G. , Holbrook, C. , Ozias‐Akins, P. , & Wang, J. (2020). Mapping quantitative trait loci (QTLs) and estimating the epistasis controlling stem rot resistance in cultivated peanut (*Arachis hypogaea*). Theoretical and Applied Genetics, 133(4), 1201–1212. 10.1007/S00122-020-03542-Y 31974667

[tpg270089-bib-0014] Pandey, M. K. , Agarwal, G. , Kale, S. M. , Clevenger, J. , Nayak, S. N. , Sriswathi, M. , Chitikineni, A. , Chavarro, C. , Chen, X. , Upadhyaya, H. D. , Vishwakarma, M. K. , Leal‐Bertioli, S. , Liang, X. , Bertioli, D. J. , Guo, B. , Jackson, S. A. , Ozias‐Akins, P. , & Varshney, R. K. (2017). Development and evaluation of a high‐density genotyping ‘Axiom_*Arachis*’ array with 58 k SNPs for accelerating genetics and breeding in groundnut. Scientific Reports, 7(1), Article 40577. 10.1038/srep40577 28091575 PMC5238394

[tpg270089-bib-0015] Spadaro, D. , & Gullino, M. L. (2005). Improving the efficacy of biocontrol agents against soilborne pathogens. Crop Protection, 24(7), 601–613. 10.1016/J.CROPRO.2004.11.003

[tpg270089-bib-0017] Tamba, C. L. , & Zhang, Y. M. (2018). A fast mrMLM algorithm for multi‐locus genome‐wide association studies. *bioRxiv*. 10.1101/341784

[tpg270089-bib-0018] Uffelmann, E. , Huang, Q. Q. , Munung, N. S. , Vries, D. J. , Okada, Y. , Martin, A. R. , Martin, H. C. , Lappalainen, T. , & Posthuma, D. (2021). Genome‐wide association studies. Nature Reviews Methods Primers, 1(1), Article 59. 10.1038/s43586-021-00056-9

[tpg270089-bib-0019] Veerendrakumar, H. V. , Kiranmayee, B. , Vasanthi, R. P. , Kumar, N. A. R. , Pandey, M. K. , & Sudini, H. K. (2024). Enhancing phenotyping accuracy for stem rot disease through advanced oxalic acid assay in groundnut. BMC Plant Biology, 24(1), Article 1042. 10.1186/s12870-024-05706-0 39497083 PMC11536941

[tpg270089-bib-0020] Vikas, V. K. , Pradhan, A. K. , Budhlakoti, N. , Mishra, D. C. , Chandra, T. , Bhardwaj, S. C. , Kumar, S. , Sivasamy, M. , Jayaprakash, P. , Nisha, R. , Shajitha, P. , Peter, J. , Geetha, M. , Mir, R. R. , Singh, K. , & Kumar, S. (2022). Multi‐locus genome‐wide association studies (ML‐GWAS) reveal novel genomic regions associated with seedling and adult plant stage leaf rust resistance in bread wheat (*Triticum aestivum* L.). Heredity, 128(6), 434–449. 10.1038/s41437-022-00525-1 35418669 PMC9177675

[tpg270089-bib-0021] Yan, L. , Song, W. , Wang, Z. , Yu, D. , Sudini, H. K. , Kang, Y. , Lei, Y. , Huai, D. , Chen, Y. , Wang, X. , Wang, Q. , & Liao, B. (2023). Dissection of the genetic basis of resistance to stem rot in cultivated peanuts (*Arachis hypogaea* L.) through genome‐wide association study. Genes, 14(7), 1447. 10.3390/genes14071447 37510351 PMC10378806

