# Supplementary material for: Dissecting the genomic regions, candidate genes and pathways using multi‐locus genome‐wide association study for stem rot disease resistance in groundnut
Source: Plant Genome. 2025 Aug 11;18(3):e70089. doi: 10.1002/tpg2.70089 (PMC12340334; doi:10.1002/tpg2.70089)
Supplement: Supplementary file 1 — Includes the information on biotic stress related genes identified through GWAS (Sheet 1). KEGG IDs and Enzyme ID of identified genes derived using Phytozome 13 database (Sheet 2). Sequence of markers used in Axiom_Arachis array for validation (sheet 3). List of genotypes used for validation (Sheet 4). [file TPG2-18-e70089-s001.docx]

**Supplementary File**

Includes the information on biotic stress related genes identified through GWAS (Sheet 1). KEGG IDs and Enzyme ID of identified genes derived using Phytozome 13 database (Sheet 2). Sequence of markers used in Axiom_*Arachis* array for validation (sheet 3). List of genotypes used for validation (Sheet 4).
